# Long term outcomes of antiretroviral therapy in a large HIV/AIDS care clinic in urban South Africa: a prospective cohort study

**DOI:** 10.1186/1758-2652-12-38

**Published:** 2009-12-17

**Authors:** Ian M Sanne, Daniel Westreich, Andrew P Macphail, Dennis Rubel, Pappie Majuba, Annelies Van Rie

**Affiliations:** 1 Clinical HIV Research Unit, Dept of Medicine, University of the Witwatersrand, Johannesburg, South Africa; 2 Department of Epidemiology, University of North Carolina, Chapel Hill, NC, USA

## Abstract

**Background:**

Clinical, immunologic and virologic outcomes at large HIV/AIDS care clinics in resource poor settings are poorly described beyond the first year of highly active antiretroviral treatment (HAART). We aimed to prospectively evaluate long-term treatment outcomes at a large scale HIV/AIDS care clinic in South Africa.

**Methods:**

Cohort study of patients initiating HAART between April 1, 2004 and March 13, 2007, and followed up until April 1, 2008 at a public HIV/AIDS care clinic in Johannesburg, South Africa. We performed time to event analysis on key treatment outcomes and program impact parameters including mortality, retention in care, CD4 count gain, virologic success and first line regimen durability.

**Results:**

7583 HIV-infected patients initiated care and contributed to 161,000 person months follow up. Overall mortality rate was low (2.9 deaths per 100 person years, 95% CI 2.6-3.2), but high in the first three months of HAART (8.4 per 100 person years, 95% CI 7.2-9.9). Long-term on-site retention in care was relatively high (74.4% at 4 years, 95%CI 73.2-75.6). CD4 count was above 200 cells/mm^3 ^after 6 months of treatment in almost all patients. By the fourth year of HAART, the majority (59.6%, 95%CI 57.8-61.4) of patients had at least one first line drug (mainly stavudine) substituted. Women were twice as likely to experience drug substitution (OR 1.97, 95% CI 1.80-2.16). By 6 months of HAART, 90.8% suppressed virus below 400 copies. Among those with initial viral suppression, 9.4% (95% CI 8.5-10.3%) had viral rebound within one year of viral suppression, 16.8% (95% CI 15.5-18.1) within 2 years, and 20.6% (95% CI 18.9-22.4) within 3 years of initial suppression. Only 10% of women and 13% of men initiated second line HAART.

**Conclusion:**

Despite advanced disease presentation and a very large-scale program, high quality care was achieved as indicated by good long-term clinical, immunologic and virologic outcomes and a low rate of second line HAART initiation. High rates of single drug substitution suggest that the public health approach to HAART could be further improved by the use of a more durable first line regimen.

## Background

Infection with human immunodeficiency virus (HIV) affects over 33 million people globally[1]. Global access to highly active antiretroviral therapy (HAART) has increased dramatically, but the majority of those in need remain untreated, especially in sub-Saharan Africa. With more than five million individuals living with HIV and AIDS, South Africa has the largest population of HIV-infected individuals[1,2]. Following the political commitment made to include HAART in the Comprehensive Care, Management and Treatment program of the public health sector from April 2004 onwards, South Africa now has the largest number of people receiving HAART in the world[3]. Scale-up of HAART has however been slower than anticipated, and the treatment gap remains in excess of 500,000 individuals.

Antiretroviral treatment roll-out clinics in sub-Saharan Africa have achieved clinical, immunologic and virologic outcomes in the first year of HAART comparable to those observed in developed countries [4-14]. Little is known about the longer term outcomes of these large-scale roll-outs. A recent review of ART programs in sub-Saharan Africa suggested that, due to loss to follow up and death, only 60% of those initiating HAART are still on treatment by the end of year two[15]. There is thus legitimate concern that a focus on numbers of people initiated on HAART may compromise quality of care.

In this study of a large (> 7,500 patients) and rapidly expanding (> 200 new patients each month since 2004) clinic in Johannesburg, South Africa, we evaluated the outcomes of the first four years of activity (> 160,000 person months follow-up) of a South African Department of Health program by comprehensively assessing five key parameters: mortality, loss to follow-up, CD4 count gain viral suppression, and durability of first line HAART regimen.

## Methods

### Study site and inclusion criteria

The Themba Lethu Clinical Cohort is a prospective clinical cohort of adults initiating HAART in Johannesburg, South Africa. The program is funded by the South African National and Gauteng Department of Health, with support from Right to Care funded by USAID and PEPFAR. The Themba Lethu Clinic at the regional Helen Joseph Hospital in urban Johannesburg has over 17,000 patients in care and is currently the largest single clinic providing HAART in South Africa, and one of the largest HAART clinics worldwide.

Patients were included in this study if they initiated HAART between 1 April 2004 and 31 March 2007. Patients were censored at death, loss to follow-up or on 31 March 2008, whichever came first.

### Routine HIV care, treatment and data collection

Patients are referred to the clinic from voluntary counseling and testing clinics, hospitals, prenatal care facilities, other ART clinics or by self-referral. HAART-eligible patients attend educational and adherence sessions, and are assessed by a physician prior to initiating treatment. After HAART initiation, patients are scheduled for monthly pharmacy visits, clinical visits at month 4 and every 6 months thereafter, and additional visits whenever needed. Patients receive all antiretroviral medications free of charge but were required to pay 35 Rand (about $5 US) per clinic visit prior to October 2006, after which all fees were eliminated. Patients more than three months late for a scheduled clinic or pharmacy visit are actively traced (three phone calls and a home visit if needed) to ascertain the reason for loss to follow-up.

As per the South African national guidelines, the majority of patients receive a first-line HAART regimen of stavudine (d4T), lamivudine (3TC), and either efavirenz (EFV) or nevirapine (NVP). Most pregnant women receive lopinavir boosted with ritanovir (LPVr) in place of EFV or NVP. Second-line HAART consists of AZT, didanosine (DDI), and LPVr.

Clinical and laboratory (including CD4 count, viral load and hemoglobin) data are collected at all scheduled visits, except for viral load which is not collected at baseline. All data were stored in the TherapyEdge-HIV™ (Associated Biological Systems - SA) database, and analyzed in SAS v9.1.3 (SAS Institute, NC, USA).

### Data quality

Results were regularly controlled for quality by a senior nurse or data quality assurance manager. Duplicate entry of CD4 counts, viral loads, and hemoglobin from the National Health Laboratory Service database into the TherapyEdge-HIV database demonstrated high quality of data entry as 98.8% of values matched between the two databases.

### Definitions

Patients more than three months late for a scheduled visit, and on whom the tracing team was unable to gain information were categorized as *lost to follow-up*. *Retention into care *refers to patients known to be alive and receiving HAART at Themba Lethu Clinic at the end of follow up (March 31, 2008). Viral suppression was defined as achieving viral load below 400 copies per ml. *Initial virologic success *was defined as achieving a viral suppression within six months of HAART initiation in patients with a viral load measured between 30 days and 6 months of HAART. *Viral rebound *was defined as two consecutive (or one if no additional measurement was available) viral load measurements greater than 400 copies/ml, or one viral load greater than 5000 following initial virologic success. *Body mass index *(BMI) was measured in kg/m^2 ^and categorized as underweight (BMI < 18.5), normal (18.5 ≤ BMI < 25), overweight (25 ≤ BMI < 30), and obese (BMI ≥ 30) [16]. *Anemia *was defined as a hemoglobin (Hb) value below 13.0 g/dl (men), 12.0 g/dl (women), or 11.0 g/dl (pregnant women)[17], Hb values were down-adjusted by 0.65 g/dl because of altitude[18]. *Baseline covariates *were those collected closest to time of initiation of HAART, and no more than four months before initiation of HAART or one month after initiation.

### Statistical analysis

Baseline demographics were characterized using standard descriptive statistics. Missing baseline height (n = 48) or weight (n = 20) was assigned using median height and weight by gender. Chi-square tests were used to compare categorical variables and Wilcoxon rank-sum tests for continuous variables by category. Rates were estimated using crude Poisson models.

To examine the effect of CD4 count on mortality and loss to follow-up, we created Kaplan-Meier curves stratified by baseline CD4 counts, and used competing-risks Cox proportional hazards models [19,20] to estimate the effect of CD4 count on both death and loss to follow-up simultaneously. In addition, we used inverse probability of censoring weights to adjust estimates of mortality for loss to follow-up using measured covariates [21].

CD4 gain was assessed using two linear generalized estimating equations models with an unstructured correlation matrix. To allow the slope of CD4 increase to change over time, the first model included the main variable of months since HAART initiation, indicator variables for baseline CD4 count, and static changepoints at months 4, 10, and every 6 months thereafter (corresponding to routine clinic visits) until end of follow-up. To allow the slope to change both in time and by initial CD4 count, the second model included all terms from the first model as well as interaction terms between baseline CD4 count categories, continuous time and all static changepoints.

Time to drug substitution, initiation of second-line HAART or virologic failure was evaluated using Kaplan-Meier curves with right censoring of the latter at time of last viral load assessment and censoring at time of death and loss of follow up. Rates of changes in drug regimens and drug toxicities were assessed using Poisson models and simple descriptive statistics.

All analyses were intent-to-treat with regards to HAART, ignoring treatment interruptions. Time to loss to-follow-up was set at the last observed visit plus half the mean time between clinic visits of any kind while in care (20 days). Where CD4 counts or viral load measurements were missing, complete case analysis was performed. Multiple imputation (10 imputations) for missing CD4 counts was performed to check the assumption that complete case analysis was appropriate.

This study was reviewed by the Institutional Review Boards of the University of the Witwatersrand and the University of North Carolina at Chapel Hill.

## Results

### Baseline characteristics

Between 1 April 2004 and 31 March 2007, a total of 7583 (about 210 new patients each month) initiated first-line HAART at the clinic. Data from 7536 (99.4%) patients was available for analysis (Table 1). Forty patients were excluded due to missing demographic information and seven because of errors in recorded dates of therapy. The majority (83.8%) of non-pregnant patients initiated d4T-3TC-EFV, 7.9% initiated d4T-3TC-NVP, 2.0% initiated d4T-3TC-LPV/r, and 6.3% initiated other regimens. In contrast, most pregnant women (84.0%) initiated d4T-3TC-LPVr. Two-thirds (66.5%) of patients were women of whom 11.5% were pregnant at time of HAART initiation. Mean age was 35.4 years in women and 38.7 years in men (p < 0.0001) (Table 1). At time of ART initiation, more than half (56%) of patients were unemployed.

Patients presented with advanced HIV disease, with median baseline CD4 count of 87 cells/mm^3 ^(IQR 31-158), one third (34.4%) of patients initiated HAART at CD4 < 50 cells/mm^3^, and19.1% of patients were underweight. Men presented at an older age and with more advanced disease (lower CD4 counts, lower BMI, anemia and WHO III and IV disease) compared to non-pregnant women, and non-pregnant women presented with more advanced disease compared to pregnant women.

**Table 1:** Baseline characteristics of 7536 individuals enrolled in Themba Lethu Clinical Cohort (TLCC) by gender and pregnancy status

	Female, pregnant (n = 574)	Female, non-pregnant (n = 4437)	Male (n = 2525)	p value (non-pregnant female vs. male)
**Age (Median and IQR)**	34.0 (29.4-40.2)	34.9 (30.1-41.2)	37.2 (32.8-43.3)	< 0.0001 †
**Ethnicity**				0.5818
African or black	560 (97.6)	4253 (95.9)	2375 (94.1)	
Coloured	14 (2.4)	153 (3.5)	97 (3.8)	
Asian, White, or Missing	0 (0.0)	31 (0.7)	53 (2.1)	
**Employment**				< 0.0001
Employed, student, retired	195 (34.0)	1455 (32.8)	1086 (43.0)	
Unemployed	354 (61.7)	2619 (59.0)	1262 (50.0)	
Unknown or missing	25 (4.4)	363 (8.2)	177 (7.0)	
**BMI**				< 0.0001
< 18.5	7 (1.2)	786 (17.7)	639 (25.3)	
18.5-24.9	208 (36.2)	2369 (53.4)	1538 (60.9)	
25-29.9	230 (40.1)	829 (18.7)	279 (11.1)	
≥ 30	128 (22.3)	421 (9.5)	54 (2.1)	
Missing	1 (0.2)	32 (0.7)	15 (0.6)	
Hemoglobin*				0.7088
Normal	399 (69.5)	2095 (47.2)	1202 (47.6)	
Low	163 (28.4)	2298 (51.8)	1294 (51.3)	
Missing	12 (2.1)	44 (1.0)	29 (1.2)	
**WHO stage**				0.0003
I	427 (74.4)	1944 (43.8)	1011 (40.0)	
II	64 (11.2)	566 (12.8)	286 (11.3)	
III	74 (12.9)	1482 (33.4)	927 (36.7)	
IV	9 (1.6)	445 (10.0)	301 (11.9)	
**CD4 count**				
Median (IQR)	146 (91-192)	86 (32-157)	75 (23-146)	< 0.0025 †
Mean (95% CI)	145 (138-152)	111 (107-114)	98 (94-102)	< 0.0001 ‡
≤ 50	53 (9.2)	1370 (30.9)	950 (37.6)	< 0.0001
51-99	91 (15.9)	885 (20.0)	453 (17.9)	
100 - 199	249 (43.4)	1319 (29.7)	703 (27.8)	
200 - 349	91 (15.9)	366 (8.3)	169 (6.7)	
≥ 350	6 (1.1)	129 (2.9)	61 (2.4)	
Missing	84 (14.6)	368 (8.3)	189 (7.5)	
**Initial HAART regimen**				< 0.0001
d4T-3TC-EFV	54 (9.4)	3622 (81.6)	2211 (87.6)	
d4T-3TC-NVP	33 (5.8)	436 (9.8)	117 (4.6)	
d4T-3TC-LPVr	482 (84.0)	112 (2.5)	24 (1.0)	
Other	5 (0.9)	267 (6.0)	173 (6.9)	

All figures are expressed as number (% total), except where noted. P-values are two-sided by chi-square test of general association across all categories by sex, except † Wilcoxon rank-sum test or ‡ t-test. *Lower limit of normal hemoglobin is 12.35 g/dl for men; 11.35 g/dl for non-pregnant women; and 10.35 g/dl for pregnant women.

### Survival, loss to follow-up and retention in care

During 161,000 person-months of follow-up (mean 21.4 months, median 20.3 months), 385 (5.1%) patients died and 1234 (16.4%) patients were lost to follow up. More than one in three deaths (39.2%, n = 151) and losses to follow-up (38.9%, n = 480) occurred in the first 90 days of treatment. The crude rate of death was 2.9 deaths per 100 person-years (95% CI 2.6-3.2) overall. Early death rate was higher: 8.4 (95% CI 7.2-9.9) per 100 person-years in the first three months and 6.8 (95% CI 6.0-7.7) per 100 person-years in first six months of HAART.

**Figure 1:**
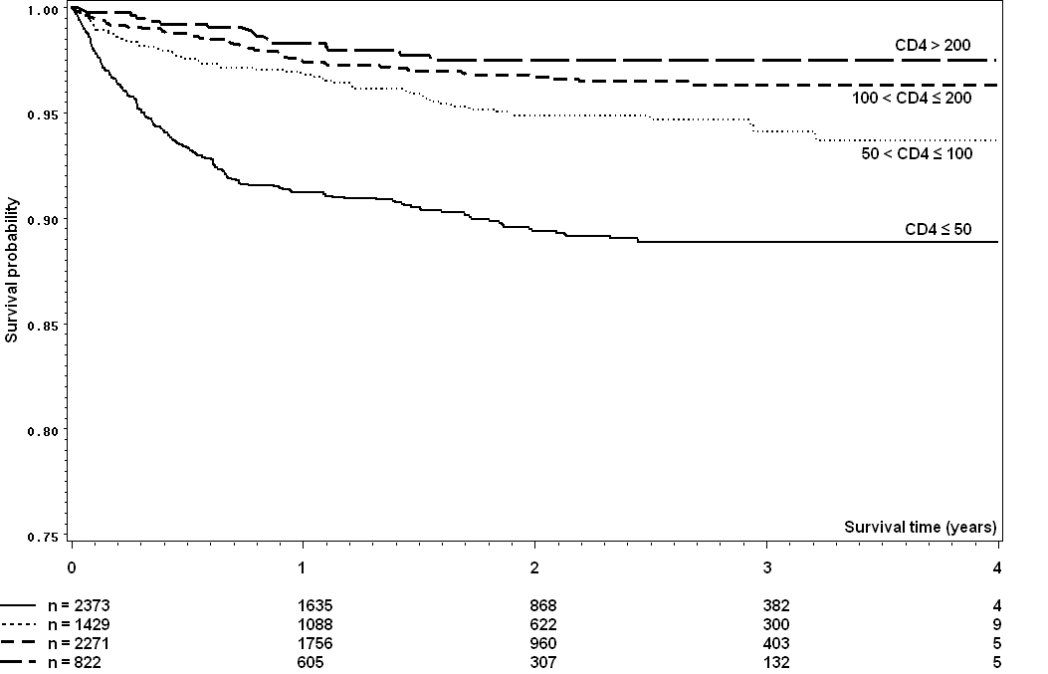
**Kaplan-Meier curves of survival by CD4 count by time since HAART initiation**. Note that the scale of the y-axis starts at 0.75 for readability.

**Figure 2:**
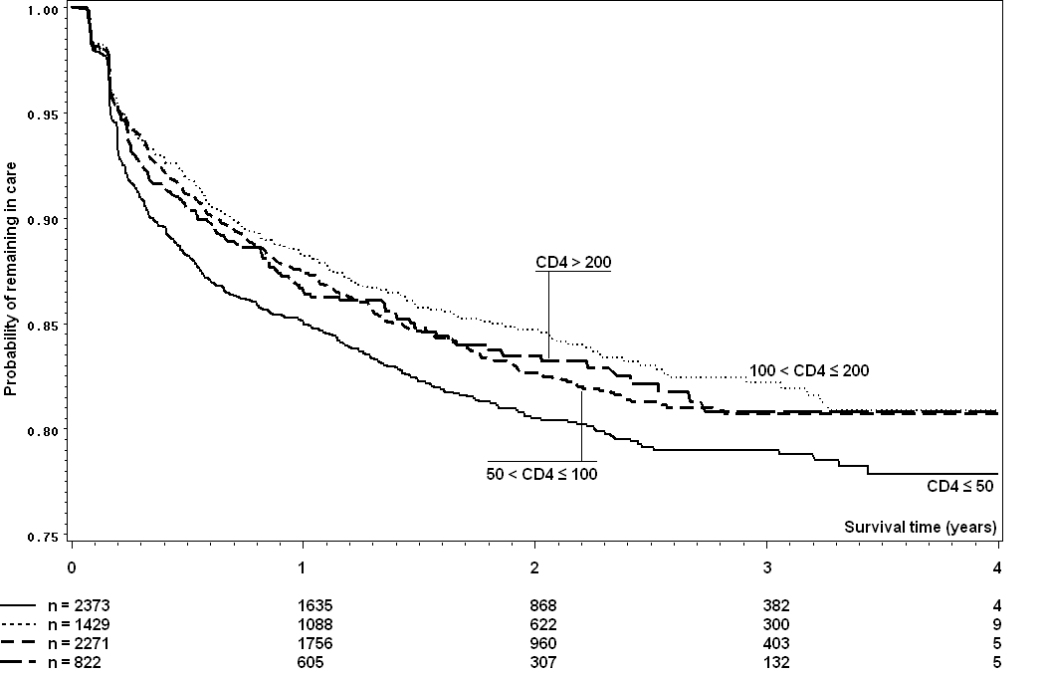
**Kaplan-Meier curves of lost to follow-up by CD4 count by time since HAART initiation**. Patients were categorized as *lost to follow-up *if more than three months late for a scheduled visit, and if the tracing team was unable to gain information. Note that the scale of the y-axis starts at 0.75 for readability.

Crude survival curves for death and loss to follow-up are shown in Figures 1 and 2. In a competing risks analysis, risk of death was almost five times higher (HR 4.87, 95% CI 2.94-8.09) among patients with baseline CD4 count ≤ 50 cells/mm^3 ^compared to those with baseline CD4 count above 200 cells/mm^3^. In contrast, baseline CD4 was only a weak predictor of loss to follow up (CD4 ≤ 50 cells/mm^3 ^compared to CD4 > 200 cells/mm^3^, HR 1.18, 95% CI 0.96-1.43). Inverse probability of censoring weighted estimates of the effect of CD4 count on mortality produced qualitatively very similar results. Multiple imputation analyses indicated that a complete case analysis did not affect the estimated effect of CD4 count on hazard of death during follow-up.

The Kaplan-Meier estimates of retention into care were 82.8% (95% CI 81.9-83.6) at 1 year, 77.5% (76.5-78.5) at 2 years, 75.2% (74.1-76.3) at 3 years and 74.4% (73.2-75.6) at 4 years. This corresponds to monthly attrition rates of 1.43 per month in the first 12 months of HAART, 0.94 per month in the first two years, 0.69 per month in the first three years, and 0.53 in the first four years of HAART.

### CD4 response

A first follow up CD4 count was available for 5601 (74.3%) patients after approximately 4 months (median 118 days, IQR 111-139 days, range 47-198 days). The observed median and mean CD4 count at four months of treatment was 206 and 227 cells/mm^3^. The observed mean four-month CD4 gain was 117 cells/mm^3 ^and differed by baseline CD4 count: 71 CD4 cells/mm^3 ^for individuals with a baseline CD4 > 200 cells/mm^3 ^compared to 122 CD4 cells/mm^3 ^for those with baseline CD4 ≤ 200 cells/mm^3 ^(p < 0.0001). The estimated mean CD4 count during the first three years of HAART by baseline CD4 count is shown in Table 2. CD4 count continued to rise at each time point, although at a slower rate after the first six months of HAART.

**Table 2:** Estimated* absolute CD4 count during first 3 years of HAART at HIV/AIDS clinic in Johannesburg, South Africa

	Months post HAART initiation						
	0	3	6	12	18	24	36
**Overall (n = 7536)**	111 (1)	196 (2)	239 (2)	289 (2)	341 (2)	372 (3)	441 (4)
**Baseline CD4 category**							
CD4 ≤ 50 (n = 2372)	23 (1)	116 (2)	167 (2)	229 (3)	284 (4)	330 (5)	349 (5)
50 < CD4 ≤ 100 (n = 1429)	77 (1)	171 (2)	214 (3)	255 (4)	298 (5)	330 (6)	377 (10)
100 < CD4 ≤ 200 (n = 2271)	150 (1)	242 (2)	284 (3)	322 (3)	367 (4)	402 (5)	428 (10)
CD4 > 200 (n = 822)	317 (6)	362 (6)	392 (6)	434 (7)	470 (8)	506 (9)	548 (20)

*Mean (standard deviation) CD4 counts were estimated by linear generalized estimating equations model using CD4 count data on patients alive and in care

### Viral suppression and viral rebound

Among individuals who had viral load measured between 30 days and six months after HAART initiation (n = 5263 or 69.8%), 90.8% suppressed virus to ≤ 400 copies/ml. Probability of initial viral suppression did not differ by baseline CD4 count. Of the 485 patients who failed to achieve initial virologic success, 294 (60.6%) achieved viral suppression during follow-up of which about half (n = 144 or 49%)) suppressed on their first line HAART regimen.

**Figure 3:**
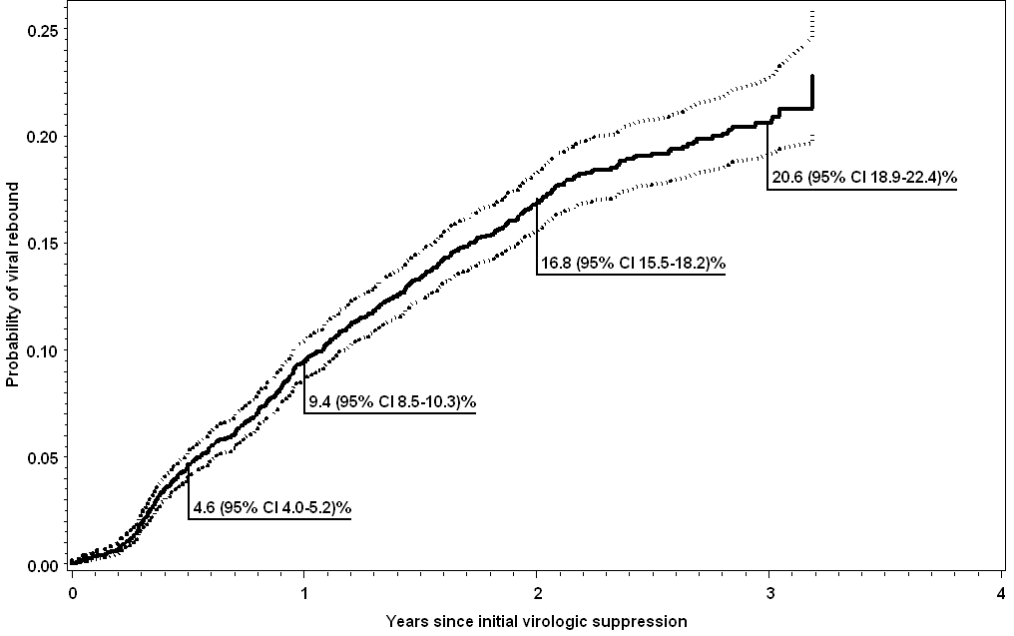
**Kaplan-Meier estimates of viral rebound among patients with initial viral suppression**. Initial virologic success is defined as viral load ≤ 400 copies/ml within six months of HAART initiation. *Viral rebound *is defined as two consecutive (or one if no additional measurement was available) viral load measurements greater than 400 copies/ml, or one viral load greater than 5000 among patients with initial virologic success.

Among those with initial virologic success, the Kaplan-Meier estimate of the proportion of patients with documented viral rebound was 9.4% (95% CI 8.5-10.3%) within one year of initial suppression, 16.8% (95% CI 15.5-18.2) within 2 years, and 20.6% (95% CI 18.9-22.4) within 3 years of initial suppression (Figure 3). Using a more conservative viral rebound definition of two viral loads > 400 copies per ml or one viral load > 5000 ml, the estimated proportion of patients with viral rebound at 3 years was 16.5% (95%CI 15.9-18.2). Only 10% of women and 13% of men initiated second line HAART.

### Durability of first line regimen

**Figure 4:**
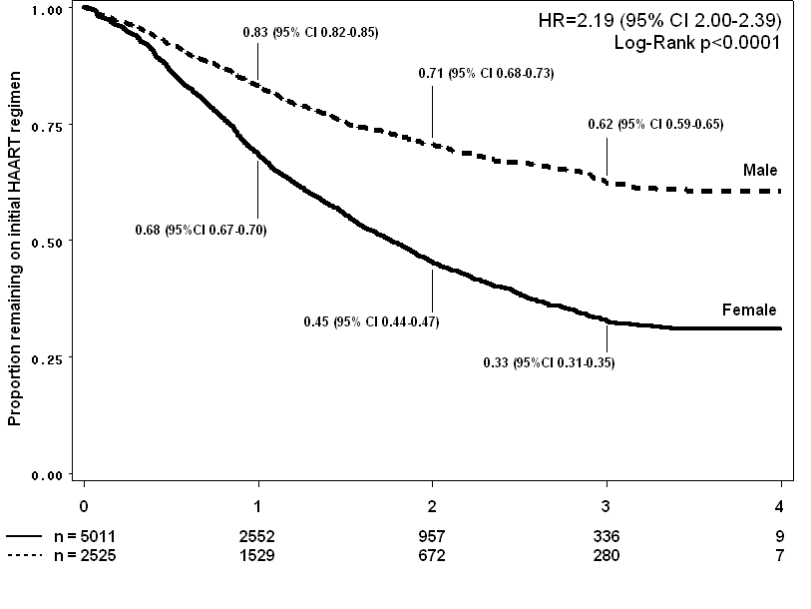
**Kaplan Meier estimates of time to first change of any kind to initial HAART regimen, separated by gender Male or Female**.

**Figure 5:**
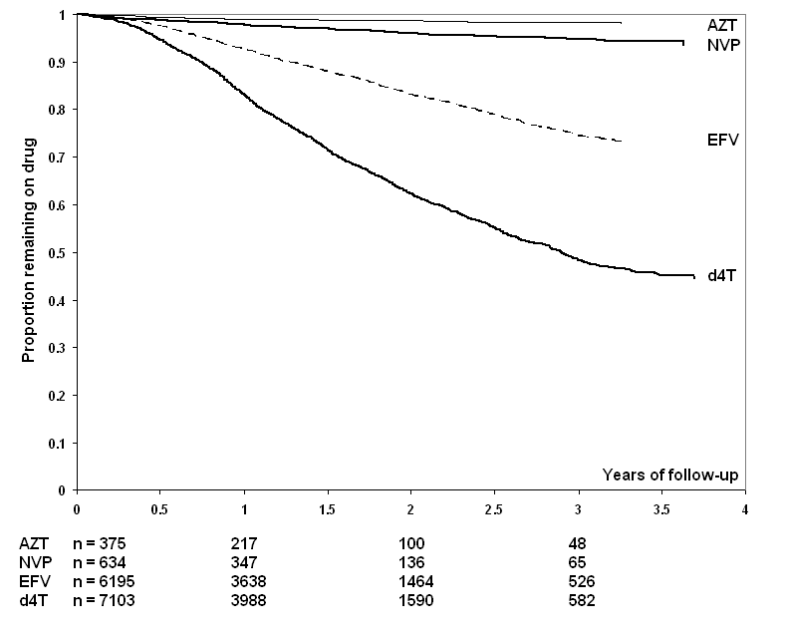
**Kaplan Meier estimates of first line regimen substitutions during first 4 years of HAART**. AZT, zidovudine; NVP, nevirapine; EFZ, efavirenz; d4T, stavudine.

A Kaplan-Meier curve of time to first drug substitution (excluding switch to second-line drug regimens) by gender is illustrated in Figure 4, and by individual drug in Figure 5. Overall, an estimated 59.6% (95%CI 57.8-61.4) of patients had experienced one or more drug substitutions by the end of four years HAART. The rate of first drug substitution in this cohort was 29.1 (95% CI 28.0-30.2) per 100 person-years. Women were twice as likely as men to experience such a drug substitution (HR 2.19, 95% CI 2.00-2.39). This association was not only due to drug substitutions due to pregnancy (excluding pregnant women, crude HR for female gender 1.97, 95% CI 1.80-2.16). The most common substitution was from d4T-3TC-EFV to AZT-3TC-EFV (38% of first drug-regimen changes).

Peripheral neuropathy, lactic acidosis or symptomatic hyperlactatemia, and lipodystrophy were the most frequent toxicities with incidence rates of 8.7 cases per 100 person-years of follow-up (95% CI 8.1-9.2) for peripheral neuropathy, 5.1 (95% CI 4.7-5.5) per 100 person-years for lactic acidosis or symptomatic hyperlactatemia, and 4.9 (4.5-5.3) cases per 100 person-years for lipodystrophy. Compared to men, women experienced peripheral neuropathy at a lower rate (HR 0.83, 95% CI 0.73-0.95), but lipodystrophy and lactic acidosis or symptomatic hyperlactatemia at higher rates than men (HR = 5.82, 95% CI 4.35-7.79 and HR = 2.68, 95% CI 2.17-3.31, respectively).

**Figure 6:**
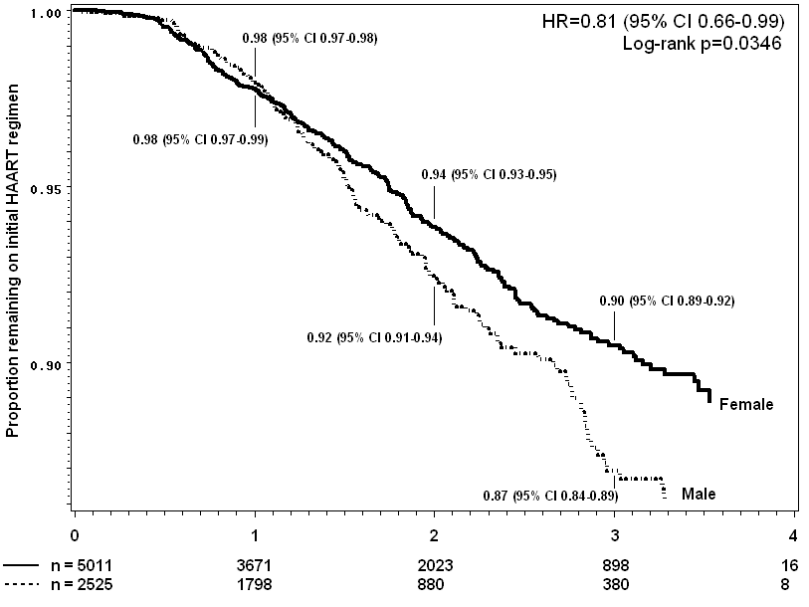
**Kaplan Meier estimates of time to initiation of second-line HAART Regimen, separated by gender male or female. Majority of patients (87%) were switched to second line following documented virologic failure**.

Second-line HAART (AZT-DDI-LPVr) was initiated by 420 individuals after a median time of 507 (IQR 309-721) days. The crude rate of initiation of second-line therapy was 3.2 (95% CI 2.9-3.6) per 100 person-years. Women were less likely to initiate second-line HAART than men (HR = 0.81, 95% CI 0.66-0.99). The proportion of patients initiating second-line HAART within the first four years of HAART was 10% for female patients and 13% for male patients (Figure 6). Second-line HAART was initiated in 81% (342 of 420) following two consecutive viral load measurements above 400 copies/ml, in 6% (27 of 420) following one viral load measurement above 400 copies/ml, and in 12% (51 of 420) on clinical or immunologic grounds, without documented loss of viral suppression.

## Discussion

Our experience demonstrates that rapid scale-up of HAART using a public health approach in sub-Saharan Africa, essential to confronting the HIV/AIDS epidemic, can be accompanied by high quality care. The analysis of 7536 patients initiating HAART in the first four years activity of a busy urban South African ART clinic demonstrated good long-term clinical, immunologic and virologic outcomes, good retention into care, but poor durability of the first line regimen due to high rates of toxicity. Our results thus provide a robust assessment of ART clinic outcomes beyond those previously reported from sub-Saharan African, which demonstrated short term success (median follow-up 12 months or less) at smaller roll-out clinics (which typically enroll 50-60 new patients per clinic per month) [5,6,8-14,22].

We observed a low overall mortality, with a crude overall death rate of 2.9 deaths per 100 person years, comparable to that observed in the Swiss HIV cohort study [23], but a relatively high death rate of 8.4 per 100 person years in the first 90 days of HAART. The overall and early death rate was comparable to that reported from two other South African clinics[22], but lower compared to those reported from other large African ART clinics, even though the level of immunosuppression in our patient population (median baseline CD4 count 87 cells/mm3 and 34.4% of patients CD4 count below 50 cells/mm^3^) was similar. For example, the death rate observed in a clinic-based ART in Lusaka, Zambia was 16.1 per 100 person years overall, and 26 per 100 person years and in the first 90 days of HAART [13]. The mortality rates in a home-based care program in Uganda was 5 per 100 person years overall and 14 per 100 person years in the first 110 days of HAART [24]. The low mortality rates observed in our cohort may, in part, be due to access to tertiary care level diagnostics and hospital care. Another possibility is that some of those lost to follow-up might have died. However, the proportion of patients with a last documented CD4 count below 50 cells/mm3 was twice as high (50% vs 25.7%) among those who died compared to those lost to follow up. Even with a CD4 count of 50 cells/mm^3 ^or less, absolute risk of death was very small among patients in care at the Themba Lethu Clinic, with only 120 deaths among 11,436 person-months in which such a CD4 count was observed. As a result, mortality rates did not change when accounting for loss to follow-up using inverse probability of censoring weights [21].

The four-year 74.4% retention into care was high compared to the rather pessimistic two-year 60% retention estimate in a recent systematic review of African ART clinics by Rosen et al [15]. The discrepancy may be due to the short (9.9 months weighted average) follow up of the studies included in the systematic review. In our population, monthly attrition rates dropped from 1.43 in the first twelve months to 0.53 in the first four years of clinic activity. Estimating retention rates based on short term follow up may thus substantially underestimate retention into care.

We observed good virologic responses to treatment and relatively infrequent first line treatment failure. By month 6 of HAART, 90.8% of patients achieved viral suppression. Viral rebound within the first 3 years of achieving suppression (corresponding to approximately the first 3.5 years of HAART) was observed in an estimated 20.6% of patients experiencing viral rebound, which is slightly lower than the 27% estimate of viral rebound observed within the same time period in treatment naïve patients in the EuroSIDA study [25]. An estimated 11% of patients switched to a second line regimen within four years of treatment. The observed rate of switching to second-line drug regimen was 3.2 per 100 person-year, substantially lower than the 13 per 100 person years clinical failure rate reported from Zambia [13], even when we analyzed data for a four-year follow-up period compared to the one-year follow-up period in Zambia. This lower switch rate in our cohort and the observation that 87% switched to second line regimen after documented virologic failure suggest that access to viral load measurements may actually reduce the rate of switching to a second line regimen.

Despite good clinical, immunologic and virologic outcomes, the durability of the first line regimen in this population was poor, with an estimated 59.6% of patients experiencing at least one drug substitution in their first line regimen (mostly d4T) in the first four years of treatment. This observation is especially alarming taken into account the public health approach, where a limited number of standardized HAART regimens are available, in contrast to the more than 20 antiretroviral drugs in industrialized countries. Female patients were twice as likely to experience drug substitutions, in part due to the higher rates of some drug toxicities and to a lesser extent to switching of antiretroviral drugs for reasons of pregnancy. These estimates for first line substitution are higher than those reported in another South African cohort, where only 28% of patients experienced substitution of at least one drug in the first 3 years of treatment [26]. The reason for this important difference is unclear. In Zambia, substitution rates in the first year of treatment were 13, 27.1 and 12 per 100 person years for d4T, AZT and NVP, respectively, which corresponds closer to the overall drug substitution rate of 29 per 100 person years overall observed in our cohort.

Our analysis has many strengths including a large sample size, resulting in robust estimates, the prospective standardized data collection, and the use of time-to-event analysis instead of cross sectional assessment, thus overcoming the limitation of overrepresentation of patients with short follow-up. Our analysis also has a number of limitations, chiefly related to the observational nature of the cohort, missing data (especially viral load measurements at baseline and in 30% of patients at follow up) and use of routine data collected from a high workload clinic. Some deaths may have been misclassified as loss to follow-up, despite the implementation of active contact tracing of patients [27]. The observation that low CD4 count was a risk factor for death but not loss to follow up suggests that the magnitude of this bias is small. The reasons for the high rates of loss to follow-up are unclear but may be related to the poor socio-economic status and high mobility of the population. Generalizability of results may be limited because of the use of data from a single clinic located at a tertiary care hospital, the dominant use of EFZ-containing first line regimens, access to viral load measurements, and the higher access to resources, both human and financial, as compared to many other sub-Saharan African settings.

## Conclusions

In this large patient cohort in Johannesburg, South Africa, we observed good long-term retention and excellent clinical, immunologic and virologic outcomes. The relatively high early mortality rate and high loss to follow up in the first months of treatment presented an important challenge to achieving the best possible HAART outcomes, and suggests the need to strengthen strategies that promote early HIV diagnosis, early access to care, and rapid initiation of HAART in the very ill patients. The high rate of single drug substitution in this population strongly suggest that the formulation of an easy-to-use, non-toxic and more durable first line regimen will be fundamental in the success of the public health approach to antiretroviral treatment in resource poor settings.

## Competing interests

The authors declare that they have no competing interests.

## Authors' contributions

AVR had full access to the all the data in the study and takes responsibility for the integrity and the accuracy of the data analysis.

*Study concept and design*: IMS, DW, APM, AVR.

*Acquisition of data*: IMS, DR, PM.

*Analysis and interpretation of data*: DW, AVR.

*Drafting of the manuscript*: DW, AVR.

*Critical revisions for important intellectual content*: IMS, DW, APM, DR, PM.

*Obtaining funding*: IMS.

*Supervision of database: *APM.

*Administrative, technical or material support*: DR, PM.

All authors read and approved the final manuscript.
